# Effects of Superfine Tricalcium Silicate Powder on the Physicochemical and Mechanical Properties of Its Premixed Cement as a Root Canal Filling Material

**DOI:** 10.3390/ma17020347

**Published:** 2024-01-10

**Authors:** Xin Duan, Yanni Tan, Dechang Zhang, Hong Wu

**Affiliations:** State Key Laboratory of Powder Metallurgy, Central South University, Changsha 410083, China; duanxin@csu.edu.cn (X.D.); zhangdechang@csu.edu.cn (D.Z.); wuhong927@126.com (H.W.)

**Keywords:** calcium silicate cement, premixed cement, root canal sealing, particle size, compressive strength

## Abstract

Calcium silicate-based cement is a promising material for filling root canals. However, it has several drawbacks to its clinical application, including difficult operation and low curing strength. In this study, we successfully prepared an ultrafine tricalcium silicate powder and investigated the effects of this ultrafine powder on the performance of the premixed tricalcium silicate cement, including the curing process, setting time, hydration products, microstructure, injectivity, fluidity, and compressive strength. The results demonstrate that the addition of ultrafine tricalcium silicate powder alters the hydration product content and product morphology of the premixed cement. By increasing the content of the ultrafine powder, the injectable property of the cement can be increased to more than 95%, the fluidity can be increased from 18 mm to 35 mm, and the curing time can be shortened from 13 h to 11 h. Notably, the addition of the ultrafine powder greatly enhances the compressive strength of the hardened cement, which increases from 20.6 MPa to 51.0 MPa. These results indicate that altering the particle size distribution of the powder is an effective method for enhancing the physicochemical and mechanical properties of tricalcium silicate cement as a root canal filling material.

## 1. Introduction

Pulpitis and periapical inflammation are common oral diseases, often caused by a microbial infection of the dental pulp and periapical tissues [1]. A root canal treatment is an effective method for treating pulp diseases and periapical inflammation. Its goal is to eliminate the bacterial infection and seal off the space to prevent bacterial growth [2]. Due to the complex structure of the root canal, tissue fluid leakage caused by poor sealing can easily lead to a secondary bacterial infection. This is a common cause of failure of root canal treatments [3].Therefore, the selection of root canal filling materials with excellent sealing properties is crucial to the success of the treatment. Water-based calcium silicate sealers, due to their excellent sealing performance, favorable antibacterial properties, and long-term dimensional stability [4,5], have revolutionized the standards and approaches to root canal filling and have shown great potential [6]. Among them, mineral trioxide aggregate (MTA) is a typical example that has shown good clinical results [7].

MTA primarily consists of tricalcium silicate (Ca_3_SiO_5_, C_3_S) and dicalcium silicate (Ca_2_SiO_4_, C_2_S) as its main powder components [8]. After being mixed with a water-based solution, MTA undergoes hydration and hardening, which provides it with sufficient compressive strength and good sealing properties to resist fluids [9,10]. Tricalcium silicate and dicalcium silicate react with water to form hydrated calcium silicate (C-S-H) and calcium hydroxide (CH). The formation of calcium hydroxide creates an environment with a high pH value, which has anti-inflammatory and antibacterial effects [11,12]. Traditional MTA requires dentists to evenly mix the powder and water solution, and then fill the root canal with the resulting paste. However, this process not only prolongs the operation time but also increases the risk of surgical failure due to uneven mixing [13,14]. Researchers have developed a premixed injectable cement based on MTA to overcome these difficulties [15]. This material replaces the water solution with a non-aqueous liquid, enabling it to be stored as a paste in a syringe without solidifying. When applied, the paste is injected into the root canal, and its non-aqueous phase undergoes a liquid exchange with the aqueous phase of the physiological fluids. This process results in the cement hydration and setting [16].

Although premixed cement solves the issue of manual mixing, it still has some drawbacks similar to those of conventional MTA. Additionally, due to differences in hydration principles, it also faces challenges such as extended setting time, limited washout resistance, and low compressive strength [17]. Researchers have primarily focused on enhancing the physicochemical and biological properties of calcium silicate-based cement through the addition of other components. Calcium chloride [18,19], magnesium phosphate [20,21], calcium carbonate [22,23], and monocalcium phosphate monohydrate [24] have all been shown to facilitate the hydration process of calcium silicate-based cement, and these results have indicated a significant reduction in setting time. In addition, shortening the setting time is generally conducive to improving the erosion resistance of the cement slurry, but most of these have a negative impact on the compressive strength of the solidified cement. Some polymer additives, such as carboxymethyl chitosan [25], gelatin [26], and sodium alginate [27,28], can effectively improve the erosion resistance of calcium silicate-based cement; these additives have good viscoelastic and hydration properties, and can reduce liquid penetration. However, although these polymers improve the erosion resistance of C_3_S cement, the setting time of C_3_S cement is inevitably prolonged due to the retarding effect of the biopolymers on the hydration process [25,28]. Although the addition of one component phase can only improve a single performance aspect, the addition of multiple components makes the research process complicated, and the results are not ideal. Thus, it is proper to use the material system with simple formulation in order to study the effect of one factor.

The particle size of the calcium silicate powder has a significant impact on the physical and chemical properties of the calcium silicate materials [29,30]. The particle size distribution of the powder can be changed with ball milling [29]. This study aims to study the effect of ultrafine tricalcium silicate powder on the physicochemical and mechanical properties of premixed tricalcium silicate cement by adjusting the distribution ratio of powders with different particle sizes. Ethylene glycol was selected as the premixed liquid phase and the liquid–solid ratio was set at 0.5 g/mL [31]. The changes in the injectable property, fluidity, setting time, and compressive strength of the premixed cement were mainly studied, and the phase composition and microstructure characteristics of the solidified cement were analyzed.

## 2. Materials and Methods

### 2.1. Preparation of C_3_S Powder

**Figure 1:**
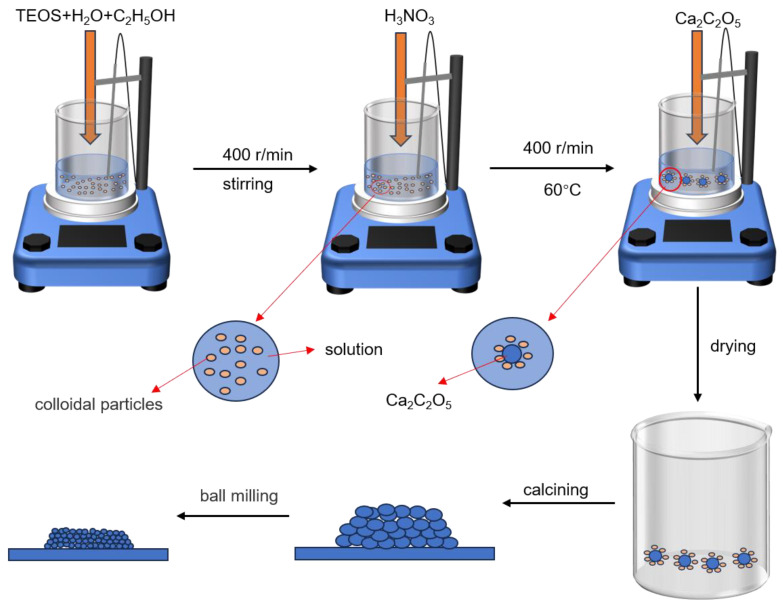
Synthesis process of C_3_S powder.

C_3_S powder was prepared using a coprecipitation method reported in the literature [32]. CaC_2_O_4_ was purchased from Shanghai Maclin Biochemical Technology Co., Ltd. (Shanghai, China), and Si(OC_2_H_5_)_4_ (TEOS), C_2_H_5_OH, and nitric acid were purchased from GuoYao Chemical Reagent Co., Ltd. (Shanghai, China). The chemical reagents used in the experiment were all analytically pure. The schematic illustration of the synthetic process of C_3_S powders is shown in Figure 1. Using CaC_2_O_4_ and TEOS as the raw materials and nitric acid as the catalyst, the initial stoichiometric ratio of CaO/SiO_2_ was set to 3. First, 0.1 mol of TEOS was added to a mixture of 40 mL deionized water and 29.3 mL of anhydrous ethanol. Then, it was continuously stirred at room temperature for 10 min to mix evenly, and the pH of the solution was adjusted to 1–2 with nitric acid. After heating the mixed solution to 60 °C, 0.3 mol of CaC_2_O_4_ as a calcium precursor was slowly added to the solution, stirred at 60 °C for about 2 h with a magnetic stirrer, and then dried in a 60 °C drying oven. The precursor powder was then calcined at 1400 °C for 6 h. Subsequently, the obtained powder was sifted through 325 mesh. The particle size of the C_3_S powder was reduced through wet ball milling (with isopropyl alcohol as the liquid phase), resulting in the production of an ultrafine powder (ultrafine powder refers to powder smaller than 10 μm).

### 2.2. Characterization of Tricalcium Silicate Powder

The phase composition of the C_3_S powder was analyzed using X-ray diffraction (XRD, D/max 2550 PC, PIGAKV, Tokyo, Japan). The target material used was Cu with a voltage of 40 KV and a current of 40 mA. The particle size distribution of the unmilled C_3_S powder was evaluated using a laser particle size analyzer (Mastersizersizer3000, Malvern Panalytical Ltd., Malver, UK) (ethanol was used as the dispersant and the powder was dispersed using an ultrasonic treatment). BM-C_3_S (tricalcium silicate after ball milling) powder was evaluated using a nanoparticle Zeta potential analyzer (Zetasizer Nano ZS, Malvern Panalytical Ltd., Malver, UK) (ethanol was used as the dispersant and the powder was dispersed using an ultrasonic treatment). The micromorphology of the C_3_S powder was analyzed using field emission scanning electron microscopy (FESEM, Tescan Mira4, Brno, Czech Republic). The sample was electrically conductive after being sprayed with platinum.

### 2.3. Preparation of Premixed Tricalcium Silicate Root Canal Sealer

Premixed cement slurry was prepared by mixing C_3_S powder with ethylene glycol (GuoYao Chemical Reagent Co., Ltd., Shanghai, China). The mass ratio of ultrafine powder to total powder is shown in Table 1. The ratio of liquid phase to powder (L/P) was set at 0.5 g/mL, which caused the premixed cement samples to have good operability. The blended sample of premixed cement was injected into a silicone mold and placed in a humid environment of 37 °C and 95% humidity for curing. Samples of premixed cement after 1 and 7 days of curing were collected and ground into powder. Crystalline phases of powders were determined using an X-ray powder diffractometer (D/max 2550 PC, PIGAKV) with a Ni filter and CuKα radiation (λ = 0.154 nm), which was generated at 40 kV and 40 mA. Samples were scanned at a range of 10°–80°, and all data were collected in a continuous scan mode at a scanning rate of 5°/min. FT-IR spectroscopic measurements were conducted using a Fourier transform infrared spectrometer (Bruker Vertex 70, Billerica, MA, USA) covering the mid-IR range (frequency: 4000–400 cm^−1^). For the measurements, the samples were diluted with KBr with a sample/KBr weight ratio of around 1/100, and compressed to tablets, which were measured relative to the KBr as a reference. Field emission scanning electron microscopy (FESEM, Tescan Mira4, Brno, Czech Republic) was used to study the fracture surface microstructure of solidified cement samples. The samples were sprayed with platinum before testing.

### 2.4. Evaluation of Injectability and Flowability

As for injectability testing, the newly prepared premixed slurry was poured into a disposable sterile syringe with an inner diameter of 0.3 mm. The premixed paste was then manually injected through the tip. Then, the injectability of the cement slurry was calculated as the percentage of the injected mass of the premixed slurry to the total mass of the original slurry. Each group was tested three times and the average was calculated (n = 3).

The flowability evaluation of the premixed slurry was determined according to ISO 6876:2012 [33]. In order to test the flowability of the premixed slurry, glass plates with a mass of 20 g (length—40 mm, width—40 mm, and height—5 mm) were prepared. The 0.05 mL premixed slurry was injected into the center of one glass plate through a syringe, then covered with another glass plate, and then loaded with 100 g weights. After 10 min, we removed the weights and measured the maximum and minimum diameters of the compression discs of the premixed slurry. If the difference between the maximum and minimum diameters was within 1 mm, the average of the two diameters was recorded. If the difference was not within 1 mm, the test was repeated. Three samples were prepared for each group and the mean value (n = 3) was calculated.

### 2.5. Setting Time

The setting time was evaluated according to ISO 6876:2012 [33]. The premixed slurry was poured into a stainless steel cylindrical mold with a diameter of 10 mm and a height of 1 mm. The samples were kept in an environment of 37 ± 1 °C and at least 95% relative humidity. A 100 ± 0.5 g Gilmore needle with a flat tip of 2.0 ± 0.1 mm in diameter was placed vertically on the surface of the sample. From the time the injection was completed until the needle tip left no trace on the sample surface, the setting time was calculated. We took 8 samples from each group (n = 8) and calculated the average value.

### 2.6. Compressive Strength

For the compressive strength test, the premixed paste was placed into a cylindrical silicone mold with a diameter of 6 ± 0.2 mm and a height of 12 ± 0.5 mm. The sample was held at 37 °C and at least 95% relative humidity for 7 days. After demolding, the sample was soaked in acetone to stop hydration. After the sample was dried, the end of each sample was ground into a flat face. After that, the compressive strength of the samples was determined using a universal testing machine (Instron 3369, Norwood, MA, USA) at a crosshead velocity of 0.5 mm/min. Four samples from each group (n = 4) were tested and the average value was calculated.

### 2.7. Washout Resistance

The freshly prepared premixed slurry was transferred to a 1 mL syringe and then extruded into a glass petri dish containing 10 mL deionized water. The washout resistance of the premixed slurry was evaluated by observing the disintegration degree of the premixed slurry at different time points.

### 2.8. Alkalizing Activity

The sample was pressed into the mold (diameter—10 mm, height—2.0 mm; n = 3 groups), and then placed in an environment of 37 °C and at least 95% relative humidity for twice the setting time [34]. The sample was then removed, immersed in 10 mL of deionized water (pH 6.5) in a polypropylene airtight container, and stored at 37 °C. The soaking water was changed at different time (5 h and 1, 3, 7, 14, and 28 days) and the pH value was measured with magnetic agitation at room temperature.

### 2.9. Statistical Analysis

Quantitative data are expressed as mean ± standard deviation (SD). All data were analyzed using SPSS 26.0 software, and the experimental results were analyzed using one-way analysis of variance (ANOVA), LSD (Least Significant Difference), and SSR (Duncan multiple range test) postmortem test. *p* < 0.05 was considered statistically significant.

## 3. Results and Discussion

### 3.1. Properties of Tricalcium Silicate Powder before and after Ball Milling

**Figure 2:**
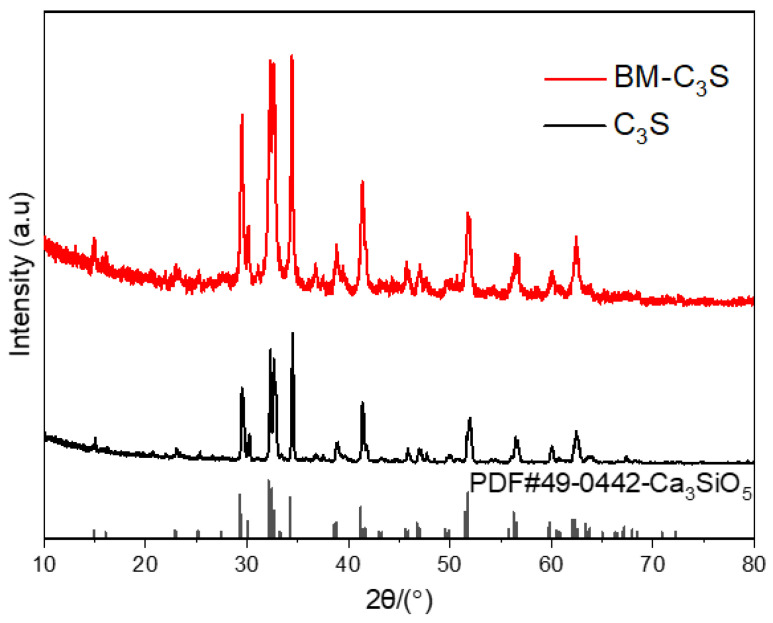
XRD patterns of the synthesized tricalcium silicate powder before (C_3_S) and after ball milling (BM-C_3_S).

**Figure 3:**
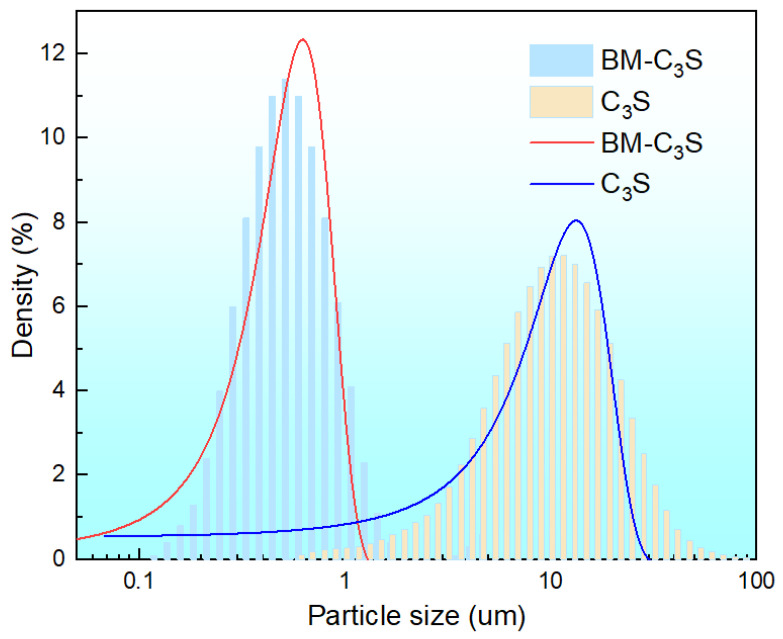
Particle size distribution of synthesized tricalcium silicate powder before (C_3_S) and after ball milling (BM-C_3_S). (The column diagram represents the particle size distribution of the powder after instrument test, and the line diagram represents the particle size distribution after Gaussian function fitting).

Figure 2 shows the XRD of the tricalcium silicate powder before and after ball milling. The phase composition of the tricalcium silicate powder remained consistent before and after ball milling. The XRD peaks with 2θ values of 29.29°, 32.12°, 34.27°, 41.2°, and 51.75° are consistent with the characteristic peaks of standard tricalcium silicate (PDF No. 49-0442) [35]. Figure 3 shows the particle size distribution of the tricalcium silicate powder before and after ball milling. The D [3, 2], D [4, 3], and Dv (50) of the powder before ball milling were 7.18 μm, 12.9 μm, 10.6 μm, respectively, and the specific surface area was 835.5 m^2^/kg. The particle size distribution of the powder before ball milling was fitted using a Gaussian function, and the average particle size of the powder was 13.27 ± 0.34 μm. The particle size decreased significantly after ball milling, with an average particle size of 496.8 nm and a peak particle size of 586.3 nm. The average particle size of the powder was 623.67 ± 10.34 nm, determined using a Gaussian function.

**Figure 4:**
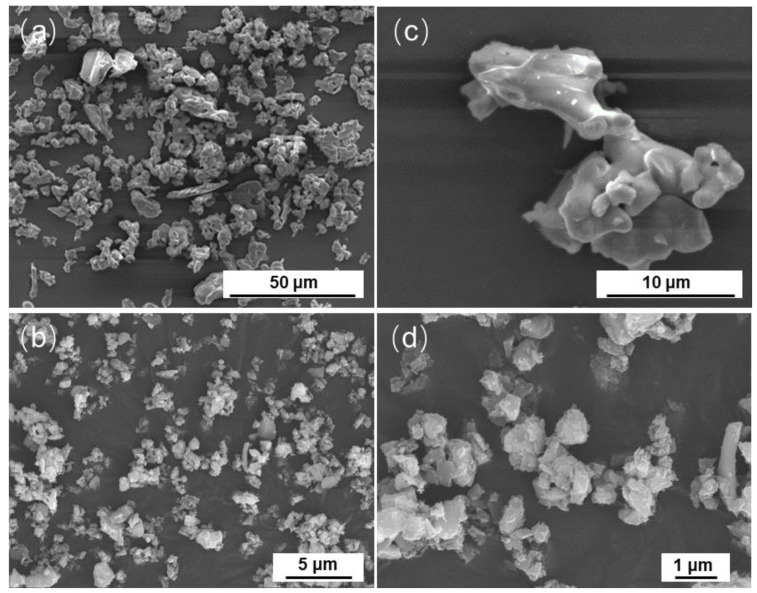
SEM images of the synthesized tricalcium silicate powder before and after ball milling. (**a**,**c**) are C_3_S; (**b**,**d**) are (BM-C_3_S).

Figure 4 shows the SEM images of the tricalcium silicate powder before and after ball milling. Figure 4a,c show that the powder before ball milling was irregularly granular, and the average particle size of the surface powder was about 10 μm, which is roughly consistent with the test results of the particle size distribution (Figure 3). Figure 4b,d show that the powder was still irregular and aggregated after ball milling, and the average particle size of the powder was about 500 nm.

C_3_S is widely believed to have the potential to become an ideal root canal filling material. However, the hydration and self-hardening rates of pure tricalcium silicate powder are very slow. The setting time and mechanical properties of injectable pastes are two critical factors for root canal treatment. The setting time is influenced by factors such as powder particle size, powder composition, solution conditions, and the L/P ratio. The smaller the particle size of the calcium silicate powder, the higher the surface area, and the faster the hydration rate [36]. Commonly used methods for preparing tricalcium silicate powder typically involve high temperatures and repeated calcination processes, which often lead to particle sizes above the micron level [37]. High-energy ball milling is an effective method for preparing nanoscale ceramic powders. The objective of this study is to obtain an ultrafine tricalcium silicate powder through wet ball milling. The results demonstrate that wet ball milling significantly reduces the particle size of the tricalcium silicate powder, making it only 1/10 the size of the initial powder, without altering its composition or morphology.

### 3.2. Hydration Phase Components of Premixed Cement Slurry

**Figure 5:**
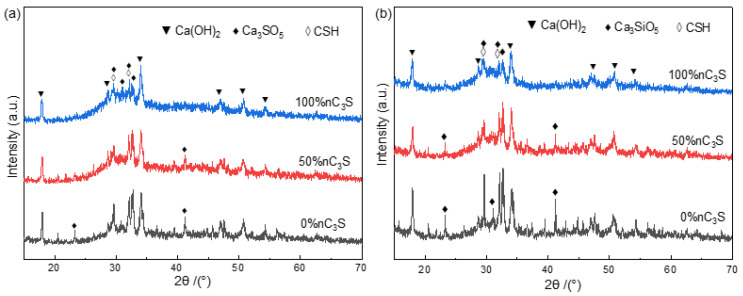
XRD patterns (**a**,**b**) of premixed cement hydrated at 37 °C for 1 day (**a**) and 7 day (**b**).

By analyzing the phase composition of the premixed cement slurry after different hydration time, the process of cement hydration after adding different amounts of ultrafine powder was studied. The XRD of the premixed cement paste after hydration for 1 day is shown in Figure 5a. The diffraction peaks of the cement were mainly composed of calcium hydroxide (PDF No.76-9571), tricalcium silicate hydrate (PDF No.33-0306), and unreacted tricalcium silicate powder (PDF No.49-0442) [38]. Figure 5b shows the XRD pattern after 7 days of hydration. The increase in storage time did not change the phase composition of the premixed paste after hydration. In the samples of the 0% nC_3_S and 50% nC_3_S, there were still the high diffraction peaks of tricalcium silicate. For the 0% nC_3_S, 50% nC_3_S, and 100% nC_3_S samples, with the increase in the ultrafine powder content, the diffraction peak intensity of the unreacted tricalcium silicate powder decreased gradually.

**Figure 6:**
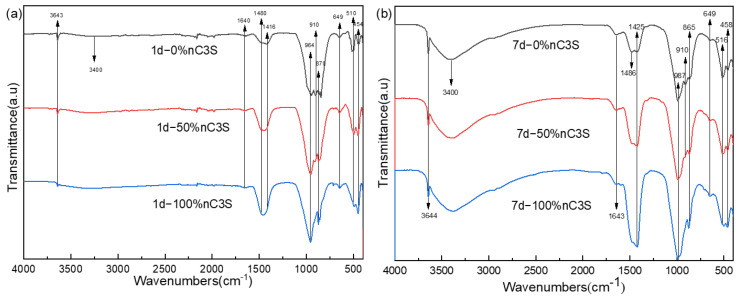
FTIR spectra (**a**,**b**) of premixed cement hydrated at 37 °C for 1 day (**a**) and 7 day (**b**).

The FTIR spectra of the premixed cement slurry samples after 1 day and 7 days of hydration are shown in Figure 6a,b, respectively. In the high-frequency range above 3000 cm^−1^, all the samples exhibit a sharp peak at 3644 cm^−1^, which corresponds to the stretching vibration of the free hydroxyl groups, indicating the presence of CH (calcium hydroxide) [39]. The intensity of this sharp peak is significantly higher in the 7-day hydrated sample compared to the 1-day hydrated sample, indicating an increased degree of hydration. Additionally, there is a broad band between 3200 cm^−1^ and 3600 cm^−1^, which is associated with the stretching vibration of O-H bonds related to the water molecules adsorbed in the C-S-H (calcium silicate hydrate) gel [38]. The absorption peak in this region is lower and less pronounced in the 1-day hydrated sample compared to the 7-day hydrated sample. All the hydrated samples exhibit similar spectra in the 1700 cm^−1^ to 1400 cm^−1^ region, with a peak at 1643 cm^−1^ corresponding to the bending vibration of the water molecules’ single bonds [40]. In the previous literature, the peak at 1425 cm^−1^ and the split peak at 1486 cm^−1^ were attributed to carbonates, suggesting the adsorption of atmospheric CO_2_ by the CH formed during hydration, and resulting in the formation of monodentate carbonate ions on the sample surface [38,39]. However, in a recent study on confined space and its effects on C-S-H structure growth, it was found that this peak decreases over time. This led to the idea that the split peaks at 1425 cm^−1^ and 1486 cm^−1^ are related to the Ca-O bonds in the C-S-H [41]. In the region below 1200 cm^−1^, the bands at 991 cm^−1^ and 870 cm^−1^ correspond to the stretching vibration of the Si-O bonds in the C-S-H. Additionally, the bands at 653 cm^−1^ and 456 cm^−1^ correspond to the stretching and bending vibrations of the Si-O-Si bonds in SiO_4_, respectively [39,41,42]. These bands gradually intensify, confirming the formation of silicate bonds. The bands at 911 cm^−1^ and 516 cm^−1^ are attributed to the characteristic peaks of unhydrated C_3_S and gradually diminish with the increase in nC_3_S content [43].

**Figure 7:**
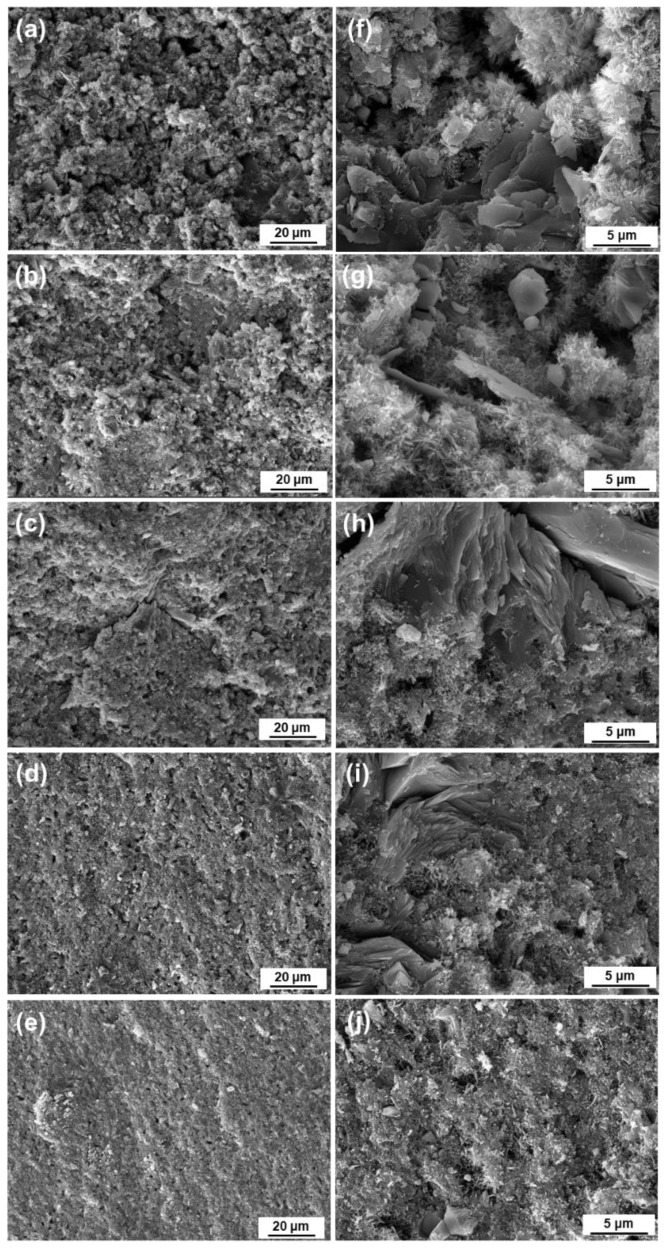
SEM images of fracture surface of premixed cements. (**a**–**e**) are the SEM images containing 0, 25%, 50%, 75%, and 100% nanometer C_3_S cement (2000×); (**f**–**j**) are the SEM images containing 0, 25%, 50%, 75%, and 100% nanometer C_3_S cement (10,000×).

The SEM images of the fracture surface of the 0% nC_3_S, 25% nC_3_S, 50% nC_3_S, 75% nC_3_S, and 100% nC_3_S after curing for 7 days are shown in Figure 7. Under a scanning electron microscope with 2000 times magnification, it can be observed from Figure 7a–e that with an increase in the content of ultrafine C_3_S powder, the fracture surface of the cement becomes more flat and gradually dense. Large pore defects can be observed on the surfaces of a and b. Obvious fracture defects exist on the surface of c, no large defects exist on the surface of d and the pores are relatively small and dispersed, and the pores on the surface of e are even smaller. Under a 10,000-fold SEM (Figure 7f–j), it can be observed that there are mainly two phases after hydration. One is a lamellar CH phase exposed in the pores, and the other is a needle-like C-S-H gel [11,44]. With an increase in the hydration time, acicular C-S-H gels are continuously formed on the surface of the C_3_S powder, and are finally interleaved to form clusters or networks covering the surface of the CH and C_3_S [35,44].

The hydration process of C_3_S typically consists of five stages: induction, acceleration, deceleration, stabilization, and the final setting [45]. When tricalcium silicate comes into contact with water, ions rapidly dissolve from the surface. C_3_S undergoes hydrolysis to form Ca^2+^, OH^−^, and H_2_SiO_4_^2−^. When the solution becomes supersaturated with calcium and silicate ions, the hydration rate reaches its maximum, and the hydration process enters the induction stage. After this stage, the cement achieves a certain level of strength. According to a simulation study on the evolution of capillary pores in cement, based on the distribution of cement particle sizes, the pore distribution for particles ranging from 3 to 40 μm shifts to 0–2 μm after 1 month of hydration. Similarly, for particles ranging from 10 to 40 μm, the pore distribution during the same period shifts to 0–5 μm [46]. This is because, under the same liquid to powder ratio, ultrafine C_3_S particles hydrate more rapidly and extensively, leading to the production of a larger amount of C-S-H gel. As the C-S-H gel grows, it fills the micropore network, resulting in a denser internal structure and a denser cement structure overall. Similar conclusions were found in this study through the investigation of the composition and microstructure of cement at various hydrationtime. With an increasing content of ultrafine powder, the cement contains more C-S-H gel phase, and the pores gradually become uniformly distributed and smaller.

### 3.3. The Injectability, Flow Property, Setting Time, and Compressive Strength of Premixed Cement

**Figure 8:**
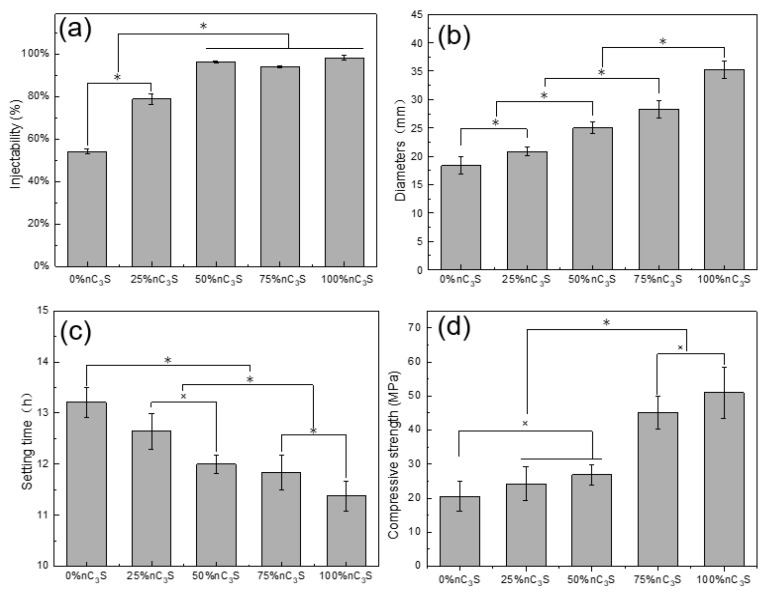
Effect of nano-powder addition ratio on injectability (**a**), flowability (**b**), setting time (**c**), and compressive strength (**d**) of premixed cement. “×” denotes values that are not significantly different. ‘‘*’’ denotes values that are significantly different.

The injectability of the premixed cement slurry is shown in Figure 8a. The injectability of the 0% nC_3_S is about 50%, and that of the 25% nC_3_S is close to 80%. The injectability of the 50% nC_3_S, 75% nC_3_S, and 100% nC_3_S are in the range of 94% to 98%, which is higher than that of the premixed calcium silicate cement without adding ultrafine powder [16,38]. With an increase in the ultrafine C_3_S powder content, the injectability of the premixed slurry is greatly improved. Good injectivity means that the premixed paste has a very high utilization rate and is also conducive to the delivery of the cement slurry to different types of cavities, which will greatly promote its application as a dental cement. During the extrusion process, the 0% nC_3_S slurry experienced significant solid–liquid separation, resulting in a poor injectable performance. The 50% nC_3_S, 75% nC_3_S, and 100% nC_3_S slurries did not appear to experience this solid–liquid separation phenomenon, and produced a complete continuous slurry with good formability. Non-premixed cement usually requires a dentist to mix the powder and liquid phase on site and then place the paste into the surgical site, which can easily lead to uneven mixing and poor sealing. Compared to non-premixed cement, the premixed preparation avoids these operations and has better handling and injection properties.

The fluidity of the premixed slurry is determined using the diameter of the pressed cement slurry [33]. The larger the diameter of the slurry, the better the fluidity of the slurry. As shown in Figure 8b, the diameters of the 25% nC_3_S, 50% nC_3_S, 75% nC_3_S, and 100% nC_3_S are greater than 20 mm, meeting the requirements of ISO 6876:2012 [33]. The diameter of the 0% nC_3_S is less than 20 mm, and the fluidity of the cement is poor. The results show that under the same L/P ratio, with an increase in the ultrafine C_3_S powder content, the flow performance of the premixed cement is significantly improved. Among them, the 100% nC_3_S has a diameter of nearly 35 mm.

The setting time of the premixed cement slurry is shown in Figure 8c. With an increase in the content of ultrafine C_3_S powder, the setting time of the premixed slurry is gradually shortened. The setting time of the 0% nC_3_S is 13.2 h, and the setting time of the 100% nC_3_S is reduced to 11.4 h. Although the addition of ultrafine C_3_S powder can shorten the setting time, the setting time of all the premixed cement slurries is longer than 10 h, which is much longer than the setting time of the water-based liquid- mixed cement slurry (2–3 h) [47]. This is because of the difference in the setting mechanism between the premixed and water-based blended cements [38,48]. Water-based cement begins to hydrate during the mixing of the powder and liquid phase, while the hydration of premixed cement is triggered after the exchange of a non-aqueous but water-miscible liquid with water molecules. As a result, the premixed cement takes more time to harden, allowing it to support the weight of the Gilmore-type indenter.

The compressive strength of the premixed cement slurry after 7 days of hydration is shown in Figure 8d. The results show that as the content of ultrafine C_3_S powder increases from 0 to 100 wt%, the compressive strength of the hardened cement increases from 20.6 MPa to 51 MPa. When the ultrafine C_3_S powder is added to the 75%, the compressive strength of the cement increases to more than 40 MPa. This is significantly higher than the compressive strength of the Bioaggregate (22 MPa, 7 days) material at 7 days, and similar to the compressive strength of the Endosequence root repair material (40–50 MPa, 7 days) at 7 days [49]. However, it is slightly lower than that of the two commercial, GMTA (55.25 ± 10.33 MPa, 7 days) and WMTA (62.04 ± 5.95 MPa, 7 days), materials [37]. It can be observed from the SEM images in Figure 7 that the addition of ultrafine powder forms a denser microstructure, thereby enhancing the compressive strength of the cement. Under the same L/P ratio, the cement with a higher concentration of ultrafine particles has a greater number of contact surfaces and reacts more rapidly than the cement with a higher concentration of coarse particles. Fine particles have smaller hydraulic radii, which increases the likelihood of pores being isolated or closed. This leads to the formation of a small interconnected pore structure, ultimately resulting in a higher compressive strength. In addition, an increase in the degree of C_3_S hydration results in the formation of more C-S-H, which may also be another factor contributing to the increase in compressive strength. This is mainly because the C-S-H gel is able to fill the micropore network, thereby reducing the number of large pores.

### 3.4. Washout Resistance of Premixed Cement

**Figure 9:**
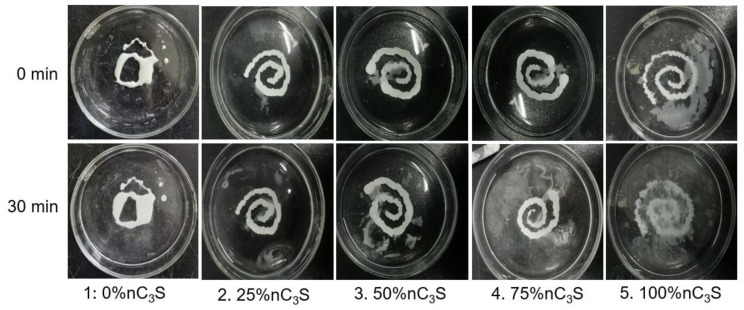
Washout resistance of premixed cement.

The erosion resistance test for the premixed cement is shown in Figure 9. The premixed slurries with 25% nC_3_S, 50% nC_3_S, 75% nC_3_S, and 100% nC_3_S can maintain a continuous form after injection into deionized water. With an increase in the ultrafine powder content, the amount of particles in contact diffusion between the slurry and the deionized water increases gradually [38]. This may be because the addition of the ultrafine powder improves the fluidity of the slurry, resulting in the poor cohesion of the slurry and more disintegration upon contact. After soaking for 30 min, it can be seen that the 100% nC_3_S cement gradually broke down, and had the highest degree of particle disintegration. This is because in the process of liquid phase exchange between the premixed slurry and the deionized water, fine particles are more easily diffused into the petri dish during the liquid phase.

### 3.5. Alkalizing Activity

The measurement results for alkalization activity are shown in Table 2. In the measurement process, all the materials alkalize the soaking medium, and the experimental results for the 1 day, 3 days, and 5 days groups have no significant difference, and are in the peak period. After 7 days, the pH value of all the samples decreases to some extent. In addition, at 5 h and 7 days, the pH values of the 75% nC_3_S and 100% nC_3_S are relatively low and significantly different from those of the 0% nC_3_S.This indicates that the increase in the ultrafine powder content has a certain effect on the pH value of the material in the early stage and after a long time of soaking, which may be due to the dense structure that slows down the release of OH^−^.

The influence of the particle size distribution of the cement powder on the physicochemical properties of a premixed cement system was studied. However, pure tricalcium silicate cement has not achieved satisfactory results in terms of setting time. Furthermore, studies that focus on the individual components cannot fully explain the curing mechanism of complex cement compositions. In the future, the incorporation of accelerators, X-ray contrast agents, and other additives will be considered to enhance the setting time and application properties of tricalcium silicate cement. Its biological properties will be studied in the future too.

## 4. Conclusions

The effects of the ultrafine C_3_S powder content on the hydration phase composition and physicochemical properties of premixed cement were studied at a 0.5 g/mL liquid to powder ratio, with ethylene glycol as the premixed liquid phase. The addition of the ultrafine C_3_S powder accelerated the hydration rate, increased the content of the hydration phase, and created a denser microstructure in the hardened cement. The higher the mass ratio of the ultrafine powder was, the better the fluidity and injectability of the premixed cement slurry, and the shorter the setting time. The compressive strength of the cement increased to 45 MPa when the ultrafine powder was added at a weight percentage of 75%. By using ultrafine powder, the compressive strength of hardened cement was increased from 20.6 MPa to 51 MPa. In conclusion, adjusting the particle size distribution of the powder can effectively enhance the physicochemical properties of the premixed cement.

## Figures and Tables

**Table 1:** Weight ratio of ultrafine powder to total powder.

Group	nC_3_S (wt.%)	C_3_S (wt.%)
0% nC_3_S	0	100
25% nC_3_S	25	75
50% nC_3_S	50	50
75% nC_3_S	75	25
100% nC_3_S	100	0

**Table 2:** Alkalization activity (mean ± SD; N = 3). The pH of the soaking water was determined after the curing sealant was soaked (+100% final curing time). Values with different superscript letters (a, b, c, d, e, f) are significantly different (*p* < 0.05).

	5 h	1 day	3 days	5 days	7 days
0% nC_3_S	10.689 ± 0.257 ^a^	10.879 ± 0.205 ^a^	10.763 ± 0.231 ^a^	10.730 ± 0.189 ^a^	10.255 ± 0.144 ^bcd^
25% nC_3_S	10.602 ± 0.210 ^abc^	10.743 ± 0.177 ^a^	10.804 ± 0.207 ^a^	10.634 ± 0.238 ^ab^	10.128 ± 0.086 ^de^
50% nC_3_S	10.617 ± 0.278 ^abc^	10.940 ± 0.237 ^a^	10.616 ± 0.297 ^abc^	10.709 ± 0.134 ^a^	10.176 ± 0.129 ^d^
75% nC_3_S	10.238 ± 0.190 ^cd^	10.736 ± 0.225 ^a^	10.737 ± 0.220 ^a^	10.574 ± 0.234 ^abc^	9.772 ± 0.208 ^ef^
100% nC_3_S	10.033 ± 0.201 ^de^	10.727 ± 0.242 ^a^	10.784 ± 0.157 ^a^	10.656 ± 0.243 ^a^	9.479 ± 0.180 ^f^

## Data Availability

The data presented in this study are available on request from the corresponding author.

## References

[1] Nair P.N.R. (2006). On the causes of persistent apical periodontitis: A review. Int. Endod. J..

[2] Siqueira Junior J.F., Rocas I.D.N., Marceliano-Alves M.F., Perez A.R., Ricucci D. (2018). Unprepared root canal surface areas: Causes, clinical implications, and therapeutic strategies. Braz. Oral Res..

[3] Siqueira J.F. (2001). Aetiology of root canal treatment failure: Why well-treated teeth can bail. Int. Endod. J..

[4] Roberts H.W., Toth J.M., Berzins D.W., Charlton D.G. (2008). Mineral trioxide aggregate material use in endodontic treatment: A review of the literature. Dent. Mater..

[5] Liu X., Chen H., Ren H., Wang B., Li X., Peng S., Zhang Q., Yan Y. (2023). Effects of ATP on the Physicochemical Properties and Cytocompatibility of Calcium Sulfate/Calcium Citrate Composite Cement. Materials.

[6] Al-Haddad A., Che Ab Aziz Z.A. (2016). Bioceramic-Based Root Canal Sealers: A Review. Int. J. Biomater..

[7] Parirokh M., Torabinejad M. (2010). Mineral Trioxide Aggregate: A Comprehensive Literature Review-Part III: Clinical Applications, Drawbacks, and Mechanism of Action. J. Endodont..

[8] Camilleri J., Montesin F.E., Brady K., Sweeney R., Curtis R.V., Ford T.R.P. (2005). The constitution of mineral trioxide aggregate. Dent. Mater..

[9] Espir C.G., Guerreiro-Tanomaru J.M., Spin-Neto R., Chavez-Andrade G.M., Camargo Villela Berbert F.L., Tanomaru-Filho M. (2016). Solubility and bacterial sealing ability of MTA and root-end filling materials. J. Appl. Oral. Sci..

[10] Solanki N.P., Venkappa K.K., Shah N.C. (2018). Biocompatibility and sealing ability of mineral trioxide aggregate and biodentine as root-end filling material: A systematic review. JCD.

[11] Camilleri J. (2011). Characterization and hydration kinetics of tricalcium silicate cement for use as a dental biomaterial. Dent. Mater..

[12] Grech L., Mallia B., Camilleri J. (2013). Characterization of set Intermediate Restorative Material, Biodentine, Bioaggregate and a prototype calcium silicate cement for use as root-end filling materials. Int. Endod. J..

[13] Luo J., Engqvist H., Persson C. (2018). A ready-to-use acidic, brushite-forming calcium phosphate cement. Acta Biomater..

[14] Salem Milani A., Radmand F., Rahbani B., Hadilou M., Haji Abbas Oghli F., Salehnia F., Baseri M. (2023). Effect of Different Mixing Methods on Physicochemical Properties of Mineral Trioxide Aggregate: A Systematic Review. Int. J. Dent..

[15] Persson C., Engqvist H. (2011). Premixed calcium silicate cement for endodontic applications: Injectability, setting time and radiopacity. Biomatter.

[16] Zhou Y., Xu C., Wang X., Dou Y., Huan Z., Chang J. (2018). Fast setting tricalcium silicate/magnesium phosphate premixed cement for root canal filling. Ceram. Int..

[17] Formosa L.M., Mallia B., Camilleri J. (2013). A quantitative method for determining the antiwashout characteristics of cement-based dental materials including mineral trioxide aggregate. Int. Endod. J..

[18] Xu C., Wen Y., Zhou Y., Zhu Y., Dou Y., Huan Z., Chang J. (2018). In vitro self-setting properties, bioactivity, and antibacterial ability of a silicate-based premixed bone cement. Int. J. Appl. Ceram. Technol..

[19] Wiltbank K.B., Schwartz S.A., Schindler W.G. (2007). Effect of selected accelerants on the physical properties of mineral trioxide aggregate and Portland cement. J. Endodont..

[20] Liu W., Zhai D., Huan Z., Wu C., Chang J. (2015). Novel tricalcium silicate/magnesium phosphate composite bone cement having high compressive strength, in vitro bioactivity and cytocompatibility. Acta Biomater..

[21] Tonelli M., Martini F., Milanesi A., Calucci L., Geppi M., Borsacchi S., Ridi F. (2019). Effect of phosphate additives on the hydration process of magnesium silicate cements: Thermal and spectroscopic characterization. J. Therm. Anal. Calorim..

[22] Wang D., Xiong C., Li W., Chang J. (2020). Growth of Calcium Carbonate Induced by Accelerated Carbonation of Tricalcium Silicate. ACS Sustain. Chem. Eng..

[23] Ashraf W., Olek J. (2018). Elucidating the accelerated carbonation products of calcium silicates using multi-technique approach. J. CO_2_ Util..

[24] Huan Z., Chang J. (2009). Novel. bioactive composite bone cements based on the beta-tricalcium phosphate-monocalcium phosphate monohydrate composite cement system. Acta Biomater..

[25] Lin Q., Lan X., Li Y., Yu Y., Ni Y., Lu C., Xu Z. (2010). Anti-washout carboxymethyl chitosan modified tricalcium silicate bone cement: Preparation, mechanical properties and in vitro bioactivity. J. Mater. Sci.-Mater. Med..

[26] Houaoui A., Szczodra A., Lallukka M., El-Guermah L., Agniel R., Pauthe E., Massera J., Boissiere M. (2021). New Generation of Hybrid Materials Based on Gelatin and Bioactive Glass Particles for Bone Tissue Regeneration. Biomolecules.

[27] Xu C., Wang X., Zhou J., Huan Z., Chang J. (2018). Bioactive tricalcium silicate/alginate composite bone cements with enhanced physicochemical properties. J. Biomed. Mater. Res. B.

[28] Ji M., Ding Z., Chen H., Peng H., Yan Y. (2019). Design of novel organic-inorganic composite bone cements with high compressive strength, in vitro bioactivity and cytocompatibility. J. Biomed. Mater. Res. B.

[29] Zheng Y., Yang X., Liu S., Xu Y., Bao S., Wang Y., Liu Y., Zhang F., Gou Z. (2022). Ball Milling Medium May Tune the Self-Curing Property and Root Canal Microleakage of beta-Dicalcium Silicate-Based Cement. Materials.

[30] Majeed R., Elnawawy H.M., Kutty M.G., Yahya N.A., Azami N.H., Kasim N.H.A., Nabhan M.S., Cooper P.R., Camilleri J., Ahmed H.M.A. (2023). Physicochemical, mechanical and biological properties of nano-calcium silicate-based cements: A systematic review. Odontology.

[31] Zhou Y., Hou D., Jiang J., She W., Li J. (2017). Molecular dynamics study of solvated aniline and ethylene glycol monomers confined in calcium silicate nanochannels: A case study of tobermorite. Phys. Chem. Chem. Phys..

[32] Wu M., Wang T., Wang Y., Li F., Zhou M., Wu X. (2018). A novel and facile route for synthesis of fine tricalcium silicate powders. Mater. Lett..

[33] (2012). Dentistry-Root Canal Sealing Materials.

[34] Zamparini F., Prati C., Taddei P., Spinelli A., Di Foggia M., Gandolfi M.G. (2022). Chemical-Physical Properties and Bioactivity of New Premixed Calcium Silicate-Bioceramic Root Canal Sealers. Int. J. Mol. Sci..

[35] Jin H., Li Y., Wang Q., Dong M., Yang M., Chen W., Wang S., Zhang H., Zheng S., Cao C.Y. (2021). A strontium and amorphous calcium phosphate dipped premixed injectable calcium silicate-based ceramic for dental root canal sealing. Ceram. Int..

[36] Zhao W.Y., Chang J. (2008). Two-step precipitation preparation and self-setting properties of tricalcium silicate. Mat. Sci. Eng. C.

[37] Lee B.-S., Lin H.-P., Chan J.C.-C., Wang W.-C., Hung P.-H., Tsai Y.-H., Lee Y.-L. (2018). A novel sol-gel-derived calcium silicate cement with short setting time for application in endodontic repair of perforations. Int. J. Nanomed..

[38] Wu M., Wang T., Zhang Y. (2021). Premixed tricalcium silicate/sodium phosphate dibasic cements for root canal filling. Mater. Chem. Phys..

[39] Zhang L., Yamauchi K., Li Z., Zhang X., Ma H., Ge S. (2017). Novel understanding of calcium silicate hydrate from dilute hydration. Cem. Concr. Res..

[40] Zhang L., Zhao C.M., Jiang Y.L., Wang Y., Yang W.S., Cheng T.X., Zhou G.D. (2018). Effect of sodium dodecyl benzene sulfonate on morphology and structure of calcium silicate hydrate prepared via precipitation method. Colloids Surf. A Physicochem. Eng. Asp..

[41] Zhou Y., Wang Z., Zhu Z., Chen Y., Zhou L., Xu L., Wu K. (2022). Time-varying structure evolution and mechanism analysis of alite particles hydrated in restricted space. Constr. Build. Mater..

[42] Yu P., Kirkpatrick R.J., Poe B., Mcmillan P.F., Cong X.D. (1999). Structure of calcium silicate hydrate (C-S-H): Near-, mid-, and far-infrared spectroscopy. J. Am. Ceram. Soc..

[43] Ren X., Zhang W., Ye J. (2017). FTIR study on the polymorphic structure of tricalcium silicate. Cem. Concr. Res..

[44] Formosa L.M., Mallia B., Bull T., Camilleri J. (2012). The microstructure and surface morphology of radiopaque tricalcium silicate cement exposed to different curing conditions. Dent. Mater..

[45] Bullard J.W., Jennings H.M., Livingston R.A., Nonat A., Scherer G.W., Schweitzer J.S., Scrivener K.L., Thomas J.J. (2011). Mechanisms of cement hydration. Cem. Concr. Res..

[46] Navi P., Pignat C. (1999). Effects of cement size distribution on capillary pore structure of the simulated cement paste. Comput. Mater. Sci..

[47] Wu M., Tao B., Wang T., Zhang Y., Wei W., Wang C. (2019). Fast-setting and anti-washout tricalcium silicate/disodium hydrogen phosphate composite cement for dental application. Ceram. Int..

[48] Takagi S., Chow L.C., Hirayama S., Sugawara A. (2003). Premixed calcium-phosphate cement pastes. J. Biomed. Mater. Res. B.

[49] Dawood A.E., Parashos P., Wong R.H.K., Reynolds E.C., Manton D.J. (2017). Calcium silicate-based cements: Composition, properties, and clinical applications. J. Investig. Clin. Dent..

